# Integrating 3HP‐based tuberculosis preventive treatment into Zimbabwe's Fast Track HIV treatment model: experiences from a pilot study

**DOI:** 10.1002/jia2.26105

**Published:** 2023-06-20

**Authors:** Munyaradzi P. Mapingure, Jennifer M. Zech, Yael Hirsch‐Moverman, Martin Msukwa, Andrea A. Howard, Tatenda Makoni, Clorata Gwanzura, Tsitsi Apollo, Charles Sandy, Godfrey N. Musuka, Miriam Rabkin

**Affiliations:** ^1^ ICAP Zimbabwe Harare Zimbabwe; ^2^ ICAP at Columbia University New York City New York USA; ^3^ Department of Epidemiology Mailman School of Public Health Columbia University New York City New York USA; ^4^ ICAP at Columbia University Pretoria South Africa; ^5^ Zimbabwe Network for People Living with HIV (ZNNP+) Harare Zimbabwe; ^6^ Ministry of Health and Child Care (MoHCC) Harare Zimbabwe; ^7^ International Initiative for Impact Evaluation (3ie) New Delhi India

**Keywords:** tuberculosis preventive treatment (TPT), differentiated service delivery, people living with HIV (PLHIV), Zimbabwe, Africa, differentiated care

## Abstract

**Introduction:**

Tuberculosis (TB) causes one‐third of HIV‐related deaths worldwide, making TB preventive treatment (TPT) a critical element of HIV programmes. Approximately 16% of people living with HIV (PLHIV) on antiretrovirals in Zimbabwe are enrolled in the Fast Track (FT) differentiated service delivery model, which includes multi‐month dispensing of antiretrovirals and quarterly health facility (HF) visits. We assessed the feasibility and acceptability of utilizing FT to deliver 3HP (3 months of once‐weekly rifapentine and isoniazid) for TPT by aligning TPT and HIV visits, providing multi‐month dispensing of 3HP, and using phone‐based monitoring and adherence support.

**Methods:**

We recruited a purposive sample of 50 PLHIV enrolled in FT at a high‐volume HF in urban Zimbabwe. At enrolment, participants provided written informed consent, completed a baseline survey, and received counselling, education and a 3‐month supply of 3HP. A study nurse mentor called participants at weeks 2, 4 and 8 to monitor and support adherence and side effects. When participants returned for their routine 3‐month FT visit, they completed another survey, and study staff conducted a structured medical record review. In‐depth interviews were conducted with providers who participated in the pilot.

**Results:**

Participants were enrolled between April and June 2021 and followed through September 2021. Median age = 32 years (IQR 24,41), 50% female, median time in FT 1.8 years (IQR 0.8,2.7). Forty‐eight participants (96%) completed 3HP in 13 weeks; one completed in 16 weeks, and one stopped due to jaundice. Most participants (94%) reported “always” or “almost always” taking 3HP correctly. All reported they were very satisfied with the counselling, education, support and quality of care they received from providers and FT service efficiency. Almost all (98%) said they would recommend it to other PLHIV. Challenges reported included pill burden (12%) and tolerability (24%), but none had difficulty with phone‐based counselling or wished for additional HF‐based visits.

**Discussion:**

Using FT to deliver 3HP was feasible and acceptable. Some reported tolerability challenges but 98% completed 3HP, and all appreciated the efficiency of aligning TPT and HIV HF visits, multi‐month dispensing and phone‐based counselling.

**Conclusions:**

Scaling up this approach could expand TPT coverage in Zimbabwe.

## 1 INTRODUCTION

Tuberculosis (TB) is the second leading cause of death from an infectious disease, causing 1.6 million deaths in 2021 [1]. Despite the scale‐up of antiretroviral therapy (ART), people living with HIV (PLHIV) are 19 times more likely to develop TB than those without HIV, and TB accounts for a substantial proportion of HIV‐related deaths [2]. Zimbabwe is a high‐burden TB/HIV country, with an estimated adult HIV prevalence of 11.9% in 2020 [3] and a TB incidence of 190/100,000 in 2021 [3]. More than half (60%) of Zimbabweans with TB are HIV positive [4]. Because TB preventive treatment (TPT) has been shown to reduce TB acquisition when combined with ART and to independently improve survival [5, 6], Zimbabwe's Ministry of Health and Child Care (MoHCC) has prioritized TPT scale‐up among PLHIV.

The MoHCC has successfully increased TPT coverage among PLHIV, with the percentage of newly enrolled ART clients receiving TPT rising from 11% in 2017 [7] to 73% in 2021 [4]. To further expand TPT coverage, MoHCC is exploring a “catch‐up” strategy to reach people who initiated ART before 2018 when TPT coverage was low, and the use of shorter TPT regimens that may increase uptake and completion.

**Figure 1:**
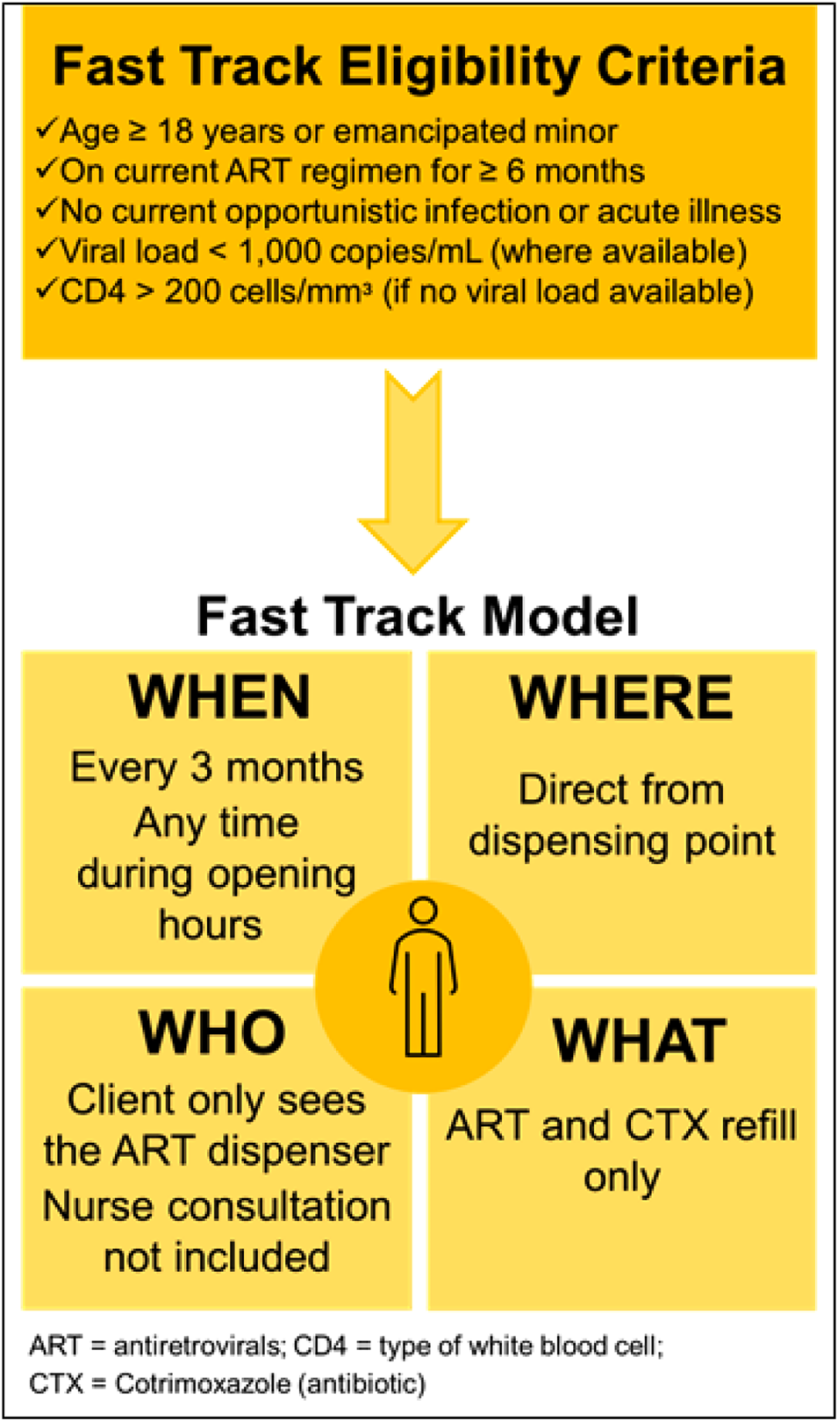
Fast Track model overview.

The catch‐up strategy recognizes that 36% of Zimbabwe's 1.1 million PLHIV on ART are enrolled in less‐intensive differentiated service delivery (DSD) models designed for people established on ART [8]. These typically involve fewer visits to health facilities, less frequent interactions with healthcare providers (HCPs), and thus fewer opportunities to initiate TPT. The largest DSD model, used by 16% of people on ART in Zimbabwe, is Fast Track, a facility‐based individual model in which clients make brief visits to health facilities every 3 months to pick up medications directly from the pharmacy/dispensing point (Figure 1). These refill visits are complemented by twice‐yearly clinical and laboratory assessments. Providing TPT in this setting recognizes the increasing interest in TPT integration into DSD models [9, 10, 11], as well as programmatic experience from Uganda [12, 13] and limited studies in Zambia [14, 15] and Zimbabwe [16], suggesting that integration of TPT into DSD is feasible and acceptable.

In addition to an intensifying focus on providing TPT for people enrolled in DSD models, MoHCC is scaling up newer TPT regimens. In 2018, the WHO endorsed using shorter TPT regimens in high‐burden settings, including 3 months of once‐weekly rifapentine and isoniazid (3HP) [17]. 3HP is safe and efficacious, can be co‐administered with dolutegravir‐based ART [18, 19], and is associated with higher completion rates [20, 21, 22]. However, 3HP is not yet widely used in Zimbabwe and has not been studied in the context of DSD models.

To assess the feasibility and acceptability of integrating 3HP into Fast Track, MoHCC, ICAP at Columbia University (ICAP) and the Zimbabwe National Network for People Living with HIV (ZNNP+) conducted a pilot project with the support of the U.S. President's Emergency Plan for AIDS Relief (PEPFAR) via the Health Resources and Services Administration (HRSA).

## 2 METHODS

### 2.1 Study setting and participants

The MoHCC provided 50 courses of 3HP for the pilot study, which was conducted at a large polyclinic in Harare. The site was selected in partnership with MoHCC and ZNNP+ based on client volume, an active Fast Track programme, and location.

We recruited a purposive sample of Fast Track clients initiating 3HP, and followed them for the duration of 3HP treatment. In addition, all HCP providing 3HP in Fast Track were invited to participate in endline in‐depth interviews (IDIs).

Fast Track clients were eligible for the study if they attended a Fast Track appointment during the recruitment period, were eligible for TPT, aged ≥18 years, enrolled in Fast Track for ≥3 months, Shona‐ or English‐speaking, had capacity for consent and were willing to participate in study activities, including audio‐recording of IDI. We aimed to recruit 50 Fast Track clients, equal numbers of men and women, and similar numbers of clients aged <30 and 30+ years. HCPs were eligible if they worked at the study site ART clinic for ≥6 months, supported Fast Track and participated in the pilot.

### 2.2 3HP pilot preparation and training

To prepare for the pilot, we developed a 3HP implementation toolkit (Appendix S1) that included job aids and monitoring and evaluation (M&E) tools. Job aids were used by providers and included a clinical algorithm to assess clients for 3HP eligibility, initiate 3HP, and monitor for adherence, side effects and TB symptoms; a pocket card to deliver messages related to 3HP initiation, adherence and side effects; an illustrated flipchart to educate clients about 3HP and adherence; a dosage chart to determine appropriate dose. M&E tools included a 3HP client management tool used by clients to note their adherence and record side effects and a call/short message service (SMS) log used by the study staff to record client interactions. The study nurse mentor conducted a 2‐day on‐site training for HCP, including nurses, medication dispensing personnel, community referral facilitators and health officers, followed by on‐site daily supportive supervision. The training covered 3HP medications and dosages, contraindications, potential side effects and adherence counselling.

### 2.3 Study activities and timeline

Using national guidelines, HCP screened Fast Track clients for TPT eligibility during routine clinic visits. Those without contraindications to 3HP were invited to participate in the study. Interested clients were referred to study staff who assessed eligibility, provided information about the study and obtained written informed consent. Clients who were not interested in participation were offered isoniazid preventive treatment (IPT) as per usual care.

#### 2.3.1 Baseline visit

Participants received a 3‐month supply of 3HP and vitamin B6 after enrolment. Ingestion of the first 3HP dose was observed by the HCP, who reviewed the quantity of pills for each medicine and the weekly dosing frequency with the participant. HCP used the job aids to provide counselling and education on adherence, side effects and TB symptoms. Clients received the 3HP client management tool to assist in documenting adherence and side effects.

Baseline data collection included interviewer‐administered surveys and IDIs to explore participants’ knowledge of TB and TPT, and goals and expectations related to integrating 3HP into Fast Track. Additionally, participant medical records were reviewed to abstract demographic and clinical information, including the date of HIV diagnosis and ART initiation, TB and TPT history, and medications.

#### 2.3.2 Remote check‐in

The study nurse mentor conducted phone check‐ins with participants at weeks 2, 4 and 8 to monitor for 3HP adherence, side effects and TB symptoms. Clients reporting side effects were referred to the clinic HCP for assessment. The nurse mentor sent manual SMS reminders prior to phone check‐ins and clinic visits, and medication reminders at intervals requested by participants. Phone calls and SMS messages were documented using the call/SMS log. The nurse mentor documented client‐reported adherence and side effects using the client tracking tool.

#### 2.3.3 Endline visit

At 3 months, participants completed an endline visit that included repeat surveys and IDIs to explore experiences related to 3HP integration into Fast Track, including adherence, side effects, perceived quality of counselling and care, convenience and acceptability. Five‐point Likert scales were used to collect information regarding client satisfaction and the use of tools and services. In addition, the 3HP client management tool was reviewed, and data were abstracted using the client tracking tool.

All HCPs participating in the pilot were invited to participate in an endline IDI. Those interested met with study staff to review study procedures and provide informed consent. IDI explored HCP experience providing 3HP and the feasibility and acceptability of integrating 3HP into Fast Track.

**Figure 2:**
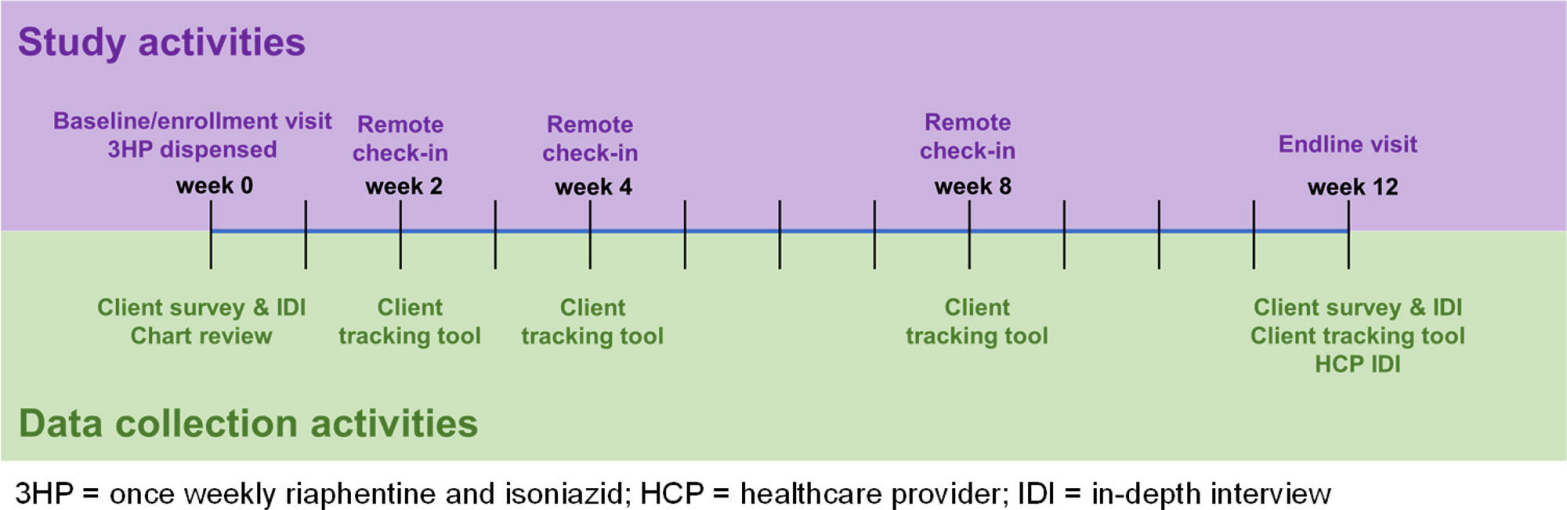
Study and data collection activities and timeline.

Study activities and timeline are summarized in Figure 2.

### 2.4 Data collection and management

A baseline chart review was conducted using a structured abstraction tool. Research assistants entered information into a paper‐based form, then uploaded data to SurveyCTO, a cloud‐based system for secure storage with internal data quality checks for valid entries, skip patterns, range checks and missing values.

Adherence, side effects and TB symptoms were documented using a paper‐based client tracking tool. The nurse mentor documented the date and outcome of each check‐in, then uploaded data to SurveyCTO.

Phone check‐ins and SMS messaging were conducted using phone numbers provided by participants. More time was spent with those who missed a dose and needed additional counselling. The nurse mentor alerted clients via SMS that they would be called the following day. If they did not pick up after two attempts, she sent an SMS asking them to alert her when ready for the check‐in.

All IDIs and surveys were conducted in private spaces by research assistants trained in human subject protections. Data were stored on encrypted tablets and computers, and devices and paper‐based tools were secured at all times.

Tablet‐based interviewer‐administered surveys with Fast Track clients were conducted in Shona (Harare's most common local language) by trained research assistants using SurveyCTO. Surveys included closed‐ended and open‐ended questions and took approximately 30 minutes.

IDIs with Fast Track clients and HCP were conducted by research assistants and took approximately 30 minutes. IDIs were audio‐recorded, transcribed, anonymized and translated into English if needed. A bilingual senior research staff member validated each IDI transcript for completeness and accuracy before analysis.

### 2.5 Data analysis

Quantitative data (client surveys, chart review, client tracking tool) were uploaded from tablets to SurveyCTO. Data were cleaned and analysed in STATA statistical software package (version 17.0). Descriptive analyses included percentages for categorical variables and median and interquartile ranges (IQR) for continuous variables.

For qualitative data, thematic analysis [23] was conducted. Each IDI transcript was summarized by a research assistant using a template developed by study investigators. A second research assistant reviewed the summary against the transcript to ensure accuracy. A matrix was developed in Microsoft Excel according to themes and sub‐themes and was used to compare themes among client and HCP participants. Investigators regularly reviewed individual summaries as well as the thematic matrix with research assistants.

### 2.6 Ethical reviews

The study was approved by the Columbia University Irving Medical Center Institutional Review Board (protocol AAAT1597), the Medical Research Council of Zimbabwe (protocol MRCZ/A/2654) and HRSA. All participants provided written informed consent prior to participation. Client participants were reimbursed $5 for their time at both the baseline and endline study visits, and received airtime equivalent to $5 at baseline for remote check‐ins. HCPs received airtime equivalent to $5 for IDI participation.

## 3 RESULTS

At the time of the study, the polyclinic provided ART to 6084 PLHIV aged ≥18 years, with an approximate ART client volume of 450 people weekly. The facility had been providing Fast Track services since 2018, and 17% of ART clients were enrolled in Fast Track.

### 3.1 Fast Track client participant characteristics

Of the 110 clients eligible for 3HP during the enrolment period, 58 were invited to participate, and eight declined. Fifty eligible adults were enrolled between April and June 2021 and followed through September 2021.

We purposively enrolled equal numbers of male and female participants. The median (IQR) age was 32 (24,41) years, 92% completed secondary education and almost half (44%) were married. All were on first‐line ART. Participants had been in Fast Track for a Median (IQR) of 1.8 (0.8,2.7) years, with 4/50 reporting previous TPT experience and 7/50 reporting a history of TB (Table 1).

**Table 1:** Baseline socio‐demographic and clinical characteristics of Fast Track client participants

Characteristics	Frequency *n* (%)
Socio‐demographic characteristics	
Median (IQR)	32 (24,41)
Male	25 (50)
Education level	
None	0 (0)
Primary	4 (8)
Secondary	41 (82)
Higher	5 (10)
Marital status	
Single (never married)	13 (26)
Married	22 (44)
Divorced/separated/widowed	15 (30)
Earnings in last month in USD	
≤100	19 (58)
101–500	11 (33)
501+	3 (9)
Clinical characteristics	
Median (IQR) years enrolled in Fast Track	1.8 (0.8,2.7)
First‐line ART regimen	50 (100)
Previous TB diagnosis	7 (14)
Previous use of TPT	4 (8)
Currently smoking	5 (10)
Membership in any HIV support group	5 (10)

### 3.2 HCP participant characteristics

We interviewed 11 HCP, including nurses, counsellors and community referral facilitators/health officers. Median (IQR) age was 43 (37,51) years and 6/11 were female. Two had worked in HIV for <5 years, while six and three had worked for 5–10 years and >10 years, respectively. Eight of 11 HCPs had been providing TPT services for >6 months. Six HCPs reported they were not trained on TPT service delivery before the pilot training was provided.

### 3.3 Baseline TB, TPT and 3HP knowledge

At baseline, most client participants were familiar with TB symptoms but less knowledgeable about TB diagnosis and treatment. Overall, participants were aware of the risks of TB, especially for PLHIV, with 90% reporting feeling vulnerable to TB.

*“They say an HIV‐positive person is more susceptible to TB infections”* (Male, 49 years)

Most participants (80%) had never heard of TPT; the few reporting TPT experience did not remember much about the treatment.

Most participants anticipated that 3HP receipt through Fast Track would be convenient. All participants were confident in their ability to adhere to 3HP as well as confident in their HCPs’ abilities to support them while taking 3HP.

“…we have always been on medication… so it is just adding a tablet on that specific day, I don't think there will be any problem” (Male, 36 years)

However, there were some concerns about drug interactions, their ability to remember once‐weekly dosing and pill burden.

Once participants received education and counselling about TB, TPT and 3HP, they were pleased to learn about TB prevention.

*“prevention is better than cure…”* (Male, 46 years)

*“ART is for life so you'll just be living positively but I won't have to worry about getting TB because I would have prevented it already”* (Female, 28 years)

Participants were happy with the education they received.

“I didn't know that TB can be prevented by taking 3HP, so I'm happy I got that support because maybe I was going to get TB without knowing it” (Male, 46 years)

### 3.4 Experience receiving 3HP in the Fast Track model

#### 3.4.1 Counselling and remote check‐in

Each participant was scheduled to receive three phone‐based check‐ins from the nurse mentor during their 3HP treatment; 150/150 of these calls were completed as planned. The nurse mentor spent an average of 10 minutes (range: 5–18 minutes) per call assessing TB symptoms, adherence and side effects, and providing counselling and support. When asked, all participants reported that TB screening and 3HP adherence and side effect monitoring were conducted at each remote check‐in.

“[the nurse] encouraged us by asking if I had taken our medication… she asked if I was experiencing some sweating and if I was coughing” (Female, 41 years)

Participants reported that receiving counselling and education at the baseline visit was the main facilitator for 3HP initiation and continued counselling and support encouraged them to complete the regimen.

*“I really felt comfortable, I felt I had all the support”* (Female, 43 years).

*“The support I got was in form of encouragement to keep on taking my pills”* (Female, 36 years)

#### 3.4.2 3HP completion and adherence

Forty‐nine of 50 participants (98%) completed a full course of 3HP. Forty‐four (88%) completed 3HP within 12 weeks, four participants completed within 13 weeks, one participant completed within 16 weeks and one stopped 3HP due to jaundice. All participants who completed 3HP “agreed” or “strongly agreed” that by taking 3HP they reduced their risk for TB.

**Figure 3:**
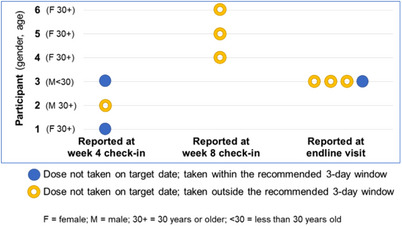
3HP doses not taken on the target date.

Except for one participant who stopped 3HP due to jaundice, all 3HP doses were taken by participants. A total of 10 doses were not taken on the target date by six participants; three were taken within the recommended 3‐day window and seven were taken outside the 3‐day window (Figure 3). No differences in sex and age were noted. Four out of the six participants who missed a dose were females and half were less than 30 years old. Participants reported missing a dose due to forgetting or travelling and not bringing the medication with them. Participants did not report any side effects when the missed dose was reported.

“I would report that I skipped a dose if I had missed it and [the nurse mentor would] call and tell me to take the medication next day same time… then the coming week go back to original day.” (Female, 40 years)

All participants noted the tools they received helped them successfully take 3HP.

*“There is a book that I would tick after taking my medication, it helped remind me so that I don't miss doses”* (Male, 29 years)

During the endline survey, participants were asked about 3HP adherence and 43 (86%) reported “very good” or “excellent” adherence within the last 30 days. Over half (58%) of participants reported no challenges with 3HP adherence. The top challenges reported were tolerability issues (24%), difficulty swallowing pills (16%) and pill burden (12%).

#### 3.4.3 Side effects

Twenty‐eight (56%) participants reported experiencing one or more side effects to the nurse mentor. The most common included headache (16%), dizziness (16%), numbness/tingling in hands or feet (14%), nausea/flu‐like symptoms (10%) and abdominal/chest pain (10%). The nurse mentor noted:

“most [side effects] were managed non‐pharmacologically” and “[some clients] did not mention [side effects] on remote check‐ins thinking that it was very minor to mention because it didn't bother them much.”

### 3.5 Experiences and views of receiving 3HP through Fast Track model

**Figure 4:**
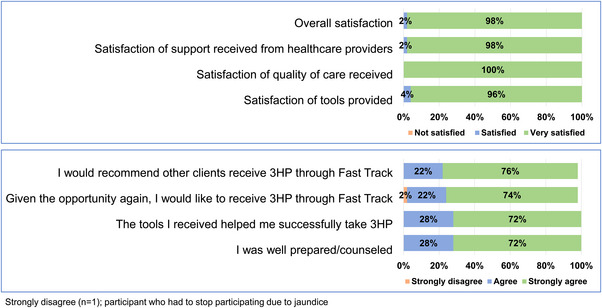
Experience and views of client participants regarding receiving TPT through Fast Track.

Almost all (96%) participants were very satisfied with receiving 3HP through Fast Track. All were satisfied with the HCP support they received while taking 3HP. All except the participant who had to stop due to jaundice (98%) “agreed” or “strongly agreed” that they would like to receive 3HP through Fast Track if prescribed again and all participants said they would recommend receipt of 3HP through Fast Track to others (Figure 4).

Table 2 presents participant perspectives on the benefits, challenges and implementation considerations regarding the integration of 3HP in Fast Track; the results are summarized by theme and include illustrative quotes from IDIs at the endline visit.

**Table 2:** Perceived benefits and challenges of the integration of 3HP in Fast Track

HCP	Clients
Benefits of integrating 3HP in Fast Track	
Combined ART and 3HP visits	
Saves time and money for clients	
“I think due to shortage of staff… and we have medication which clients take for a lesser period, I think 3HP does address those human resources challenges.” *Male health information officer*	“I usually seek leave of absence from work to collect my medication… so this was good because I get the two on the same day and saves me on transport.” *Female, 26 years*
“We were providing two services at once and also monitoring side effects was easy it was for a short period of time and also they were fewer cases [side effects] of them.” *Male nurse*	“If you are getting everything at the same time… then it saves time, it saves money also… it makes life much easier.” *Male, 49 years*
Reduces HCP workload	
“It [3HP] prevents latent TB developing to active TB. Considering that clinics are short‐staffed so if we have fewer infections also reducing the workloads.” *Male nurse*	“It has benefits for both patient and HCP because the number of visits is reduced and there will be no need to have long queues” *Male, 23 years*
Decongests clinic	
“It [integrating 3HP in Fast Track] decongested the facility thus reducing the risk [COVID] and remember we are short staffed.” *Male nurse*	“The collection of both medications at the same time… There is no crowding because patients are quickly attended to.” *Female, 32 years*
“… it also de‐congests the clinic, people won't be crowded.” *Female community referral facilitator*	“It is good because there is COVID, people will not overcrowd at the facility they will be coming back after 3 months” *Male, 19 years*
Treatment‐related benefits	
Shorter treatment duration and combined use of ART and 3HP	
“3HP is good because patients are taking it for a short time.” *Female counsellor*	“Taking them [ART and 3HP] at the same time made it easy for me rather than taking 3HP in the morning and then ART at night. I was told that I could take both at the same time.” *Female, 22 years*
Reduced pill burden/frequency (weekly vs. daily)	
“It reduces the pill burden among clients and TB among the clients.” *Male health information officer*	“It is actually good that they are taken once a week and not every day.” *Male, 23 years*
“They [3HP] prevented patients from contracting TB, pill burden was reduced and also less side effects.” *Female nurse*	“The doses are convenient, we weren't taking 3HP every day… I liked that a lot because I don't like the idea of taking pills.” *Male, 41 years*
Monitoring adherence and side effects in stable Fast Track clients	
“We can successfully monitor our client and… not waste their time because we will be aware they are on Fast Track.” *Male counsellor*	“They assess [adherence] well and they encourage us to communicate our problems.” *Female, 40 years*
“They had no problem with taking the pills and then also taking ARVs… We gave them cards to tick every time they take 3HP.” *Female counsellor*	“They [HCP] may ask the remaining number of pills, that way they can tell whether you will be taking medication as prescribed.” *Female, 26 years*
Treatment‐related challenges	
Fear of side effects and drug interactions	
“Barriers are those that come from the misinformation that will be spreading in community so a willing patient may end up being skeptical and afraid to take pills.” *Female community referral facilitator*	“I was afraid I was going to develop skin problems like rash but the nurses reassured me that if I develop any new problems I was supposed to report immediately.” *Female, 46 years*
	“…you will also be concerned about possible drug interactions of 3HP and your usual drugs [ART].” *Female, 38 years*
Increasing pill burden (size/number)	
	“My concern was around the number of pills that I was supposed to ingest at once, they were too many for me… I was comparing with what we were used to.” *Female, 24 years*
Stigma	
“There is also even stigma among the patients, willing patients won't want to be seen with pills that are being ridiculed by other patients saying they cause you to develop rash.” *Female community referral facilitator*	“Stigma is there because you can tell someone; s/he can tell you that these pills don't work. You will get hurt… Stigma is there but I continued taking my medicine.” *Female, 55 years*
“Other challenges include stigma and discrimination after other people discover that a person has TB, they say ‘do not go near him.’” *Female counsellor*	“I experienced it [stigma] where I live because I told my friend and my friend was overwhelmed and he told someone who is a whirlwind who could not keep his mouth shut.” *Male, 46 years*

Benefits highlighted by both clients and HCP included the efficiency of combined visits; treatment‐related factors; and the ability to monitor 3HP adherence and side effects in stable clients. Combined ART and 3HP visits were perceived to save clients time and money, reduce HCP workload and decongest the clinic. Treatment‐related benefits included a short treatment duration of 3HP and the ability to take 3HP and ART together, which was perceived to be easier. Furthermore, clients and HCP perceived the once‐weekly dosing as reducing the TPT pill burden. The ability to monitor adherence and side effects of both treatments concurrently was noted as another advantage.

Challenges of integrating 3HP in Fast Track included treatment‐related factors and stigma. Clients and HCP discussed fears of 3HP side effects and potential drug interactions with ART. In addition, some clients noted that despite the shorter duration and frequency of 3HP, the number and large size of the pills during the once‐weekly administration was prohibitive. Clients and HCP reported stigmatization of people with TB as another challenge.

## 4 DISCUSSION

Zimbabwe's MoHCC has prioritized the expansion of TPT coverage among PLHIV. One approach is to provide TPT to clients enrolled in DSD models in addition to those initiating ART, and provide short‐course regimens that simplify TPT delivery. We aimed to assess the feasibility and acceptability of integrating a short‐course TPT regimen, 3HP, into Fast Track.

### 4.1 Feasibility

We found that HCP and clients perceived integration of 3HP into Fast Track at a high‐volume urban health facility (HF) in Zimbabwe likely to be feasible, which we defined as the extent to which a new programme can be successfully implemented within a particular setting and a certain population [24]. HCP reported that the training, job aids and tracking tools were practical and effective. Sensitization activities increased interest in and demand for TPT, and clients were able to adhere to treatment, report side effects and participate in remote check‐ins. The use of mobile phone follow‐up allowed clients to continue in their current DSD model without additional HF visits and associated travel costs and enabled close monitoring and support. All scheduled calls were completed, and the study nurse mentor was able to effectively assess adherence, TB symptoms and side effects as well as provide counselling when needed. While 56% of clients reported some side effects, these were largely mild and only one participant had to discontinue TPT.

These findings align with other studies, including one in urban Zambia designed to integrate 6 months of IPT into a Fast Track model, using monthly phone appointments with community healthcare workers (CHWs) who provided adherence counselling and monitored side effects [25]. TPT completion rates in this project were high (90.2%) although only 65.9% of clients participated in all phone appointments. A 2016 study from Uganda reported a TPT completion rate of 72% among PLHIV in a community‐based DSD model compared to 53% in conventional facility‐based models [13]. A more recent study of TPT integration into various DSD models in Uganda also showed high completion rates [12].

Side effects from 3HP have been reported in clinical trials [20, 26, 27] and specifically in PLHIV with 3.4% of participants discontinuing treatment due to an adverse drug reaction [28] compared to 2% (1/50) in our study. A study in Pakistan found that 1% of clients receiving 3HP reported side effects [22]. Another study in Pakistan integrated 3HP into prison systems and adverse events resulted in 8.6% of participants discontinuing 3HP [29]. In the Uganda study, 36% of those who completed IPT reported one or more side effects [13], while 8.1% of clients enrolled in the TPT study in Zambia reported side effects to CHWs during phone check‐ins [25].

It is important to note that our study explored the feasibility of integrating 3HP, a 3‐month TPT regimen, while the African studies referenced above examined the integration of a 6‐month course of IPT into DSD models. Shorter TPT regimens have been shown to facilitate completion [30, 31], and the shorter course of 3HP is likely easier for both clients and HCP to integrate into existing treatment models. Our study also leveraged the time of a study nurse mentor, highlighting the importance of staff with time and training to support clients during their course of TPT. The experience of using CHW in Zambia and HCP in Uganda suggests that these tasks can successfully be performed by existing HCP.

### 4.2 Acceptability

For this study, we defined acceptability as a multi‐faceted construct that reflects the extent to which people receiving an intervention consider it to be appropriate and satisfactory, based on anticipated or experienced cognitive and emotional responses to the intervention [32]. In our study, we saw high rates of satisfaction among clients receiving 3HP through Fast Track (96%) and HCP involved in delivering it (100%).

Clients and HCP in our study especially liked the efficiency of combined clinical visits which saved clients time and money and reduced HCP overwork and clinic congestion. Additionally, it enabled HCP to monitor adherence and side effects of ART and 3HP concurrently. Many clients highlighted the ability to take 3HP and ART together, which helped with adherence, and appreciated the once‐weekly administration instead of an additional daily pill. However, for some clients, the addition of a once‐weekly dose was challenging, as was the increased pill burden. We found that receipt of adequate counselling and education at baseline was the main facilitator for 3HP initiation, and that continued counselling and support motivated participants to complete the full regimen.

Overall, our findings align with experiences in other settings, in which implementers and clients endorse the integration of TPT into DSD models. In the Ugandan study described above, the odds of TPT completion were 2.6‐fold higher in clients receiving HIV treatment in a DSD model than in those receiving non‐differentiated HIV care [13]. A recent study from Zimbabwe found that policymakers, implementers, funders and PLHIV enrolled in community‐based group DSD models were confident that TPT delivery could be integrated into community‐based DSD models [16]. A 2020 perspective paper by Boyd et al. noted that reaching the goal of providing TPT to PLHIV requires providing TPT not only to PLHIV initiating ART but also to PLHIV considered stable on ART and receiving care in DSD [14].

### 4.3 Scalability and sustainability

In Zimbabwe, CHWs support HIV service delivery through adherence counselling and conducting client follow‐up. In this pilot, we made use of a nurse mentor to ensure consistent and timely delivery of all remote check‐ins. Based on the results from this pilot and additional pilot studies of 3HP implementation in other DSD models, 3HP is being rolled out nationally in Zimbabwe and remote follow‐up by CHWs is now recommended in Zimbabwe's Operational and Service Delivery Manual for HIV. This experience in Zimbabwe demonstrates the potential for scalability in other countries that use CHWs to support HIV service delivery.

### 4.4 Strengths and limitations

Strengths of our study included the use of mixed‐methods, data collection at various points of implementation and a structured, rigorous approach to documenting client experience, which resulted in rich data that can be triangulated to better inform our questions about the feasibility and acceptability of 3HP integration into Fast Track. This study has immediate policy relevance to expanding TPT coverage for PLHIV in Zimbabwe.

Our study had some limitations. It was a small pilot study at one urban HF, which limits the generalizability of results. We used a full‐time study nurse mentor to support HCP and conduct remote follow‐up; using HCP only may be challenging for large health facilities. However, in this proof‐of‐concept study, we found that remote check‐ins were not too time‐consuming (average 10 minutes each) and saved time for clients and HCPs overall. It may also be feasible to utilize CHWs to support remote check‐ins [25] and/or to use automated rather than manual SMS reminders. Additionally, participants were provided with airtime to participate in remote check‐ins.

## 5 CONCLUSIONS

Using Fast Track to deliver 3HP was feasible and acceptable for adult PLHIV who were stable on ART. Some toxicity and tolerability challenges were reported but 98% of participants completed 3HP, none wished for additional HF visits, and all appreciated the efficiency of remote counselling and side effect monitoring. Scaling up 3HP for PLHIV in Fast Track has the potential to expand TPT coverage in Zimbabwe.

## COMPETING INTERESTS

The authors declare no competing interests.

## AUTHORS’ CONTRIBUTIONS

MPM, MR, JMZ, CG and CS conceptualized the study. All authors participated in the study design and planning. MPM led the data collection team, JMZ and YH‐M led the data analysis, with support from MPM and other team members. MPM, JMZ and YH‐M wrote the first draft of the manuscript and all authors read and approved the final manuscript.

## FUNDING

This project was supported by the U.S. President's Emergency Plan for AIDS Relief, through the Health Resources and Services Administration (HRSA) of the U.S. Department of Health and Human Services (HHS) under grant UJ7HA31180, Optimizing Momentum toward Sustainable Epidemic Control.

## DISCLAIMER

This information or content and conclusions should not be construed as the official position or policy of, nor should any endorsements be inferred by HRSA, HHS or the U.S. Government.

## Supporting information

Supplementary information

## Data Availability

The data that support the findings of this study are openly available in FigShare at https://figshare.com/articles/journal_contribution/Integrating_3HP‐based_tuberculosis_preventive_treatment_into_Zimbabwe_s_Fast_Track_HIV_treatment_model_Experiences_from_a_pilot_study/21598839.
