# Supplementary figures and images for: Integrating 3HP‐based tuberculosis preventive treatment into Zimbabwe's Fast Track HIV treatment model: experiences from a pilot study
Source: J Int AIDS Soc. 2023 Jun 20;26(6):e26105. doi: 10.1002/jia2.26105 (PMC10281638; doi:10.1002/jia2.26105)

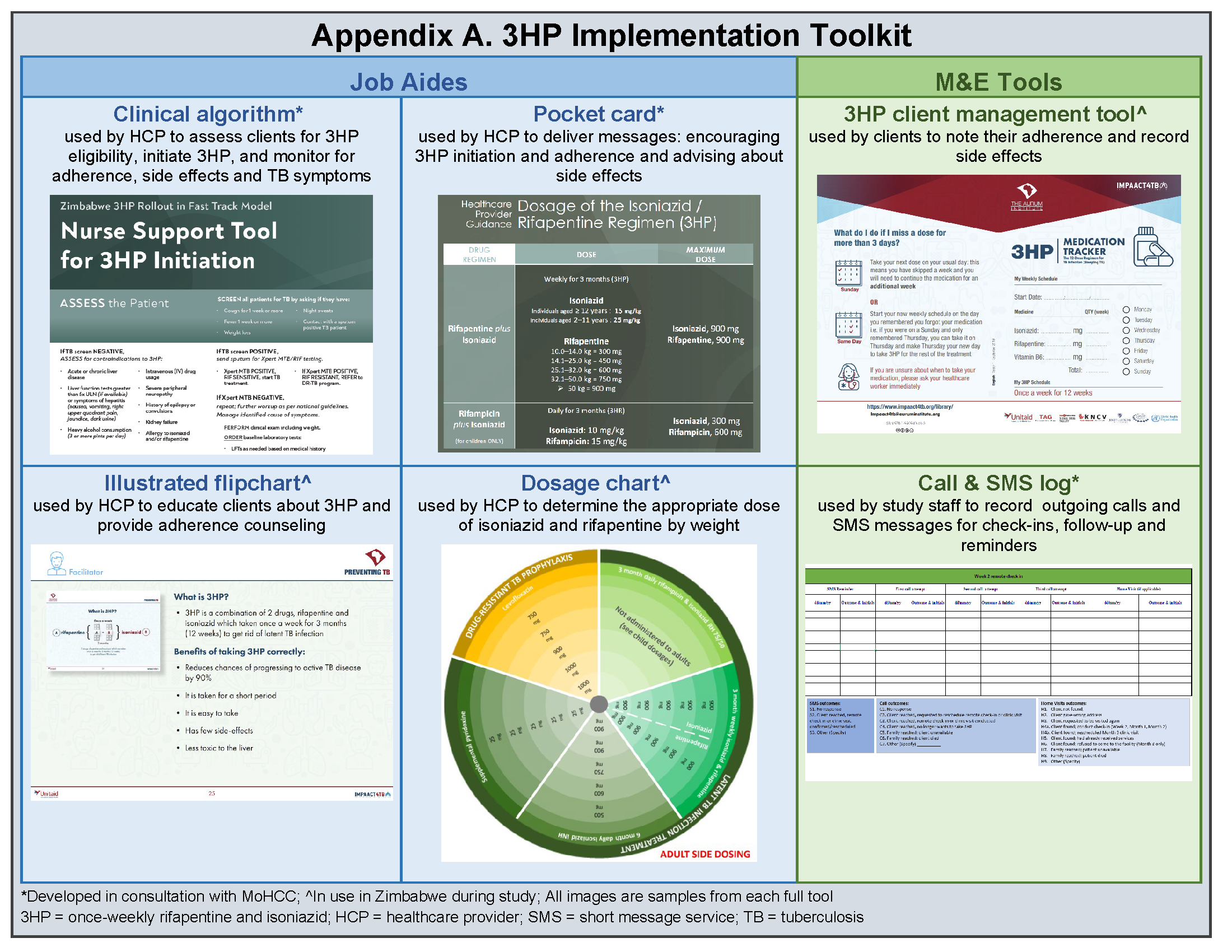

Supplement: Supplementary file 1 — Supplementary information [file JIA2-26-e26105-s001.png]
